# Unusual drug-resistant psychotic state with epilepsy manifestations, cognitive deterioration and muscular atrophy - case report

**DOI:** 10.1192/j.eurpsy.2021.648

**Published:** 2021-08-13

**Authors:** M. Bazhmin

**Affiliations:** Faculty Of Medicine, TechnionShaar Menashe Mental Health Center, Shaked, Israel

**Keywords:** Epilepsy, psychosis, cognitive deterioration, tic disorder

## Abstract

**Introduction:**

This article will focus on the case of patient whose disease manifested after episode of dehydration at 16 years of age and within 8 years led to his death. During the eight years of illness, the patient suffered from polymorfic psychotic states accompanied by various types of epileptic seizures, including absence and grand-mal seizures, resistant to drug therapy. In addition, he suffered from multiple motor disorders, skeletal and mimiс muscle atrophy, as well as progressive cognitive decline from very high level of cognitive functions to level of moderate deterioration. Despite repeated evaluations, there was no unequivocal diagnosis of his disorder.

**Objectives:**

Male, born in 1994, without a known hereditary pathology in the field of neurology or psychiatry. Pregnancy and childbirth proceeded without features and the early stages of cognitive-motor development were marked as normative, with the exception of a single epileptic seizure at the age of 3 years (according to the description of the parents). Until the age of 16, the patient was not under the supervision of a neurologist or psychiatrist, developed on a par with his peers, successfully attended school with high marks in the exact sciences, and went in for sports.

**Methods:**

Case report

**Results:**

Case report

**Conclusions:**

A patient is considered with a non-standard course of psychosis and epilepsy, which was accompanied by multiple neurological and psychiatric symptoms.In Israel there are only 13 patients with a resemble clinical picture and there is no diagnosis or group of diagnoses in ICD, DSM or any neulogical classificatin tha can describe his disease.

